# Analysis of vaccinia virus temperature-sensitive I7L mutants reveals two potential functional domains

**DOI:** 10.1186/1743-422X-3-64

**Published:** 2006-08-31

**Authors:** Megan J Moerdyk, Chelsea M Byrd, Dennis E Hruby

**Affiliations:** 1 Department of Microbiology, Oregon State University, Corvallis, OR, 97331, USA; 2 SIGA Technologies, Inc., 4575 SW Research Way, Corvallis, OR, 97333, USA

## Abstract

As an approach to initiating a structure-function analysis of the vaccinia virus I7L core protein proteinase, a collection of conditional-lethal mutants in which the mutation had been mapped to the I7L locus were subjected to genomic sequencing and phenotypic analyses. Mutations in six vaccinia virus I7L temperature sensitive mutants fall into two groups: changes at three positions at the N-terminal end between amino acids 29 and 37 and two different substitutions at amino acid 344, near the catalytic domain. Regardless of the position of the mutation, mutants at the non-permissive temperature failed to cleave core protein precursors and had their development arrested prior to core condensation. Thus it appears that the two clusters of mutations may affect two different functional domains required for proteinase activity.

## Findings

Vaccinia virus (VV) is the prototypic member of the orthopoxviruses, a genus of large, double-stranded DNA viruses which includes the human pathogens variola virus and monkeypox virus. VV has a complex replication cycle where, as in many other viruses, proteolysis plays a key role in the maturation process. The initial step in virion assembly is envelopment of viroplasm by crescent shaped membranes to form immature virions (IV). The IVs must then undergo a series of morphological changes, including cleavage of a number of core protein precursors, to become intracellular mature virions (IMV), the first of several different infectious forms.

The product of the VV I7L open reading frame (ORF) has been shown to be the viral core protein proteinase responsible for cleavage of the major core protein precursors P4a (A10L), P4b (A3L), and P25K(L4R) [1,2]. It is a cysteine proteinase, with a catalytic triad consisting of a histidine, an aspartate and a cysteine residue [2] and cleaves its substrates at conserved AG*X motifs [3-5]. In addition to the major core protein precursors, I7L has been shown to cleave the membrane protein A17L [6] and may also be responsible for the cleavage of other viral proteins containing the AG*X motif such as A12L and G7L whose cleavage has been documented but not attributed to a particular proteinase [5,7].

In the absence of functional I7L, virion morphogenesis is irreversibly arrested after the formation of IV but prior to the formation of IMV [6,8,9]. Despite the potential importance of this enzyme, relatively little is known about the biochemistry of the cleavage reaction or the structural features which allow I7L to direct regulated catalysis. Up to this point, all attempts to produce purified, functional I7L have failed, thereby limiting progress in this area. An alternative approach for studying the I7L protein is an analysis of the existing collections of temperature-sensitive (ts) mutants. Six ts mutants from the Dales and Condit collections have been identified as I7L mutants using complementation analysis [[10] and S. Kato, T. Bainbridge, N. Moussatche, and R. Condit, personal communications]. Using the classification system proposed by Lackner et al. (with the original Dales designations in parenthesis), these are: Cts-16, Cts-34. Dts-4 (260), Dts-8 (991), Dts-35 (5804), and Dts-93 (9281). Though both collections were created by chemical mutagenesis, the Condit mutants were derived from the commonly used strain Western Reserve (WR) [11,12], while the Dales mutants were derived from the strain IHD-W, an IHD-J subtype [13].

Of the six mutants, Cts-16 has been the best studied and most frequently used, primarily as a means to establish a viral infection in the absence of functional I7L. Originally it was classified as having a wild type pattern of protein synthesis [11], although it was later shown that while the major core protein precursors are synthesized, they are not cleaved at the non-permissive temperature [14]. In Cts-16, I7L has also been shown to be stably produced at the non-permissive temperature [9] and is probably included in the core. The core protein precursors also localize normally at the non-permissive temperature [14].

Dales grouped his mutants into categories based on the apparent level of development attained as determined by electron microscopy. He classified Dts-8 as a category L mutant ("immature particles with nucleoids and defective membranes with spicules") and Dts-35 as category O ("immature normal particles and mature particles with aberrant cores") [13]. Using his classification system, Cts-16 best fits category K ("granular foci and immature particles with nucleoids but lacking internal dense material") or category L. Dales did not assign Dts-93 to a category while Dts-4 was not included in the original publication. Cts-34 has also not been described other than as an I7L mutant.

In order to further characterize these ts viruses and to determine the exact location of the mutation or mutations within the I7L ORF of each virus, genomic DNA was extracted from each virus type. The I7L ORF was PCR-amplified using the primers CB26 and CB90 [15], and the same primers used to sequence the purified PCR products. Multiple copies of the sequence of the WR I7L ORF have been deposited with GenBank [GenBank: AY49736, GenBank:AY243312, and GenBank:J03399] and were obtained for this analysis.

**Figure 1:**
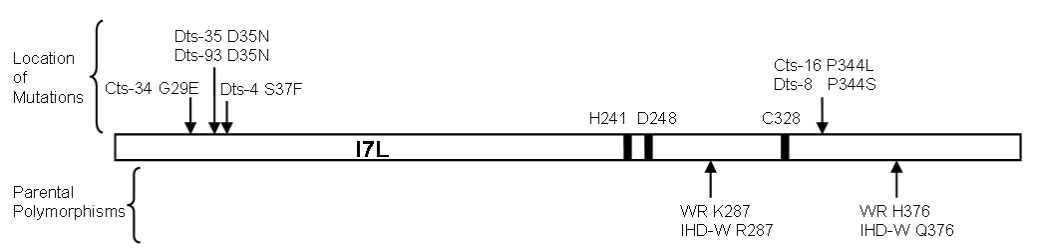
**Schematic diagram of the I7L open reading frame**. The amino acid changes found in the temperature sensitive mutants are represented above while the parental polymorphisms are given below. Black bars represent the putative catalytic triad.

Sequencing of the parental strain IHD-W revealed two differences as compared to the I7L ORF of WR with arginine instead of lysine at amino acid (aa) 287 and glutamine instead of histidine at aa376 (Figure 1). WR is reported to have either aspartate or asparagine at aa420 while IHD-W has asparagine. The I7L ORF sequence from IHD-W was identical to that of Dts-97, a mutant in the E9 ORF [S. Kato, T. Bainbridge, N. Moussatche, and R. Condit, personal communications]. When these polymorphisms are taken into account, all the I7L ts mutants contain a single amino acid change. Cts-16, as previously reported and reconfirmed in our stock, has a proline to leucine change at aa344 [9]. Cts-34 has glycine to glutamate at aa29, Dts-4 has serine to phenylalanine at aa37, Dts-8 has proline to serine at aa344, and both Dts-35 and Dts-93 have aspartate to asparagine at aa35. Interestingly, the mutations seem to form two clusters with Cts-34, Dts-4, Dts-35, and Dts-93 containing three different mutations in a stretch of nine amino acids at the N-terminal end and Cts-16 and Dts-8 representing two different mutations in a single amino acid located toward the C-terminus and just downstream of the catalytic cysteine. The possible significance of these groupings is discussed below.

Since the mutants were created by chemical mutagenesis, there is the possibility of second-site mutations that contribute to the observed phenotype. To check for this, we attempted to rescue the replication of each virus with plasmid born I7L. Using DMRIE-C (Invitrogen), BSC_40 _cells were transfected with 2 μg of either the empty vector pRB21 [16] or pI7L [8], infected at a multiplicity of infection (MOI) of 2, and incubated at the non-permissive temperature of 41C. pI7L contains the I7L ORF under the control of its native promoter and has been shown to give more efficient rescue than I7L under the control of a synthetic early/late promoter [8]. Mock transfected cells were also infected at an MOI of 2 and incubated at either 41C or the permissive temperature of 31C. The cells were harvest at 24 hours post infection (hpi), resuspended in 100 μl PBS and subjected to three freeze-thaw cycles. These lysates were titered onto confluent BSC_40 _cells in a series of 10-fold dilutions. After 48 hours of incubation at 31C, plaques were visualized by staining with 0.1% crystal violet.

**Figure 2:**
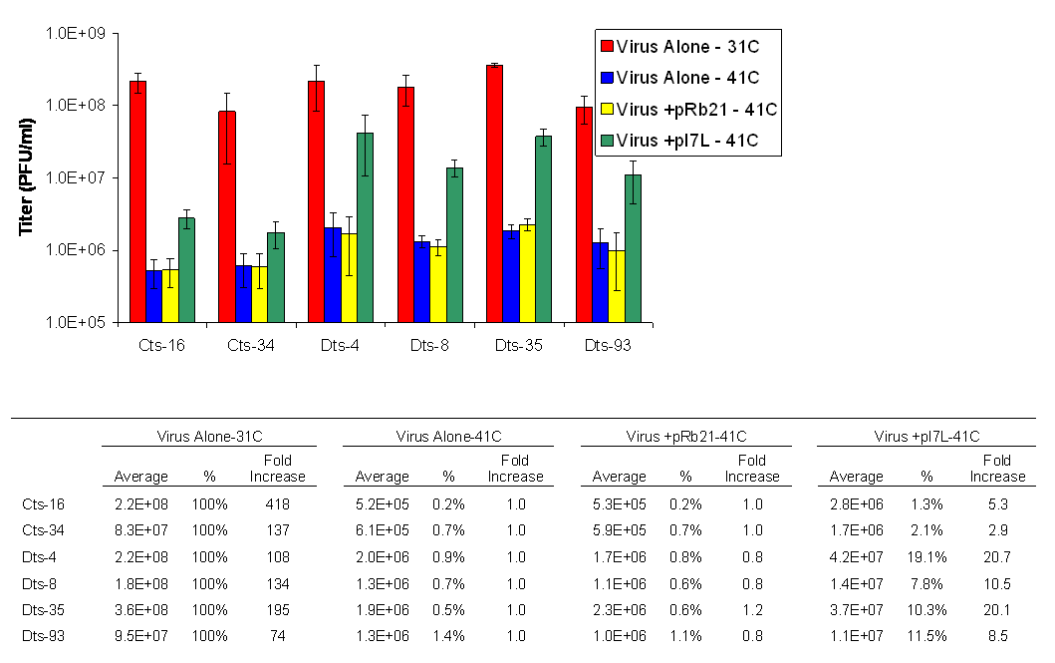
**Rescue of replication by plasmid born I7L**. BSC_40 _cells were infected/transfected as indicated and incubated at the permissive (31C) or non-permissive (41C) temperature. At 24 hours after infection, the cells were harvested and the viral titer of the diluted cell lysate was determined. Fold increase was determined by dividing the titer by the titer of virus alone at 41C. % is the percentage of the viral titer at 31C. Bars = +/-1 standard error.

All the ts mutants were rescued by the plasmid containing I7L, while transfection with an empty plasmid caused no increase in viral titer (Figure 2). This indicates that for each virus the mutation within the I7L ORF is the primary, if not only, cause of their temperature sensitive phenotype. Transfection with pI7L resulted in a 2.9 to 20.7 fold increase in viral titer over virus alone at the non-permissive temperature, causing the viruses to reach between 1.3 and 19.1% of their permissive temperature titer. Cts-34 showed the weakest rescue with a fold increase in titer only about half that of the next lowest value. However, as discussed below, its electron microscopic appearance and cleavage activity were identical to those of the other mutants, indicating that even if a second-site mutation exists, the affected protein acts with or after I7L. The degree of leakiness was low for all the mutants with the non-permissive temperature titer being 1.4% or less than that of the permissive temperature titer. As such, leakiness is not expected to have significantly affected the experiments.

**Figure 3:**
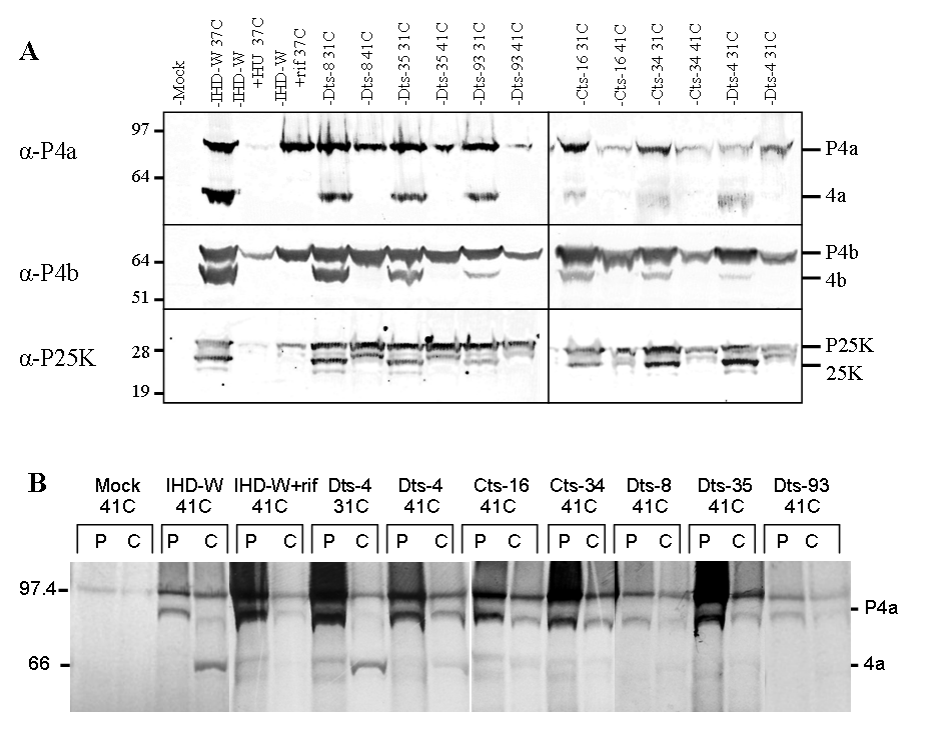
**Analysis of core protein precursor processing at the permissive (31C) and non-permissive (41C) temperatures**. (A) Infected BSC_40 _cells were incubated at the indicated temperature and harvested 24 hours after infection. Lysates were analyzed by Western blot using antisera against the indicated protein. (B) Infected BSC_40 _cells were labeled with [^35^S]-methionine and [^35^S]-cysteine for 45 minutes at 8 hours after infection. Cells were harvested after the pulse (P) and or after being chased (C) with unlabeled methionine and cysteine until 24 hours after infection. Immunoprecipitated samples were separated on a 4–12% SDS PAGE gradient gel. Rifampicin (rif) and hydroxyurea (HU) were used at final concentrations of 100 μg/ml and 8 mM respectively.

Since I7L has been implicated as the core protein proteinase, it was of interest to see if the mutants were all defective in core protein precursor cleavage. Cleavage of the core protein precursors P4a, P4b, and P25K, products of the A10L, A3L and L4R ORF's respectively, was initially assessed by western blot. BSC_40 _cells were infected at an MOI of 5, incubated at the appropriate temperature and harvested at 24 hpi. 100 μg/ml rifampicin (Boehringer-Manheim) and 8 mM hydroxyurea (applied one hour prior to infection) were used where needed. Cell pellets were resuspended in 50 μl of buffer and subjected to three freeze/thaw cycles. Aliquots of lysate were boiled with sample buffer and separated on 4–12% SDS PAGE gradient gels for P4a and P4b detection and 12% SDS PAGE gels for P25K detection. Membranes were incubated with a 1:1000 dilution of the appropriate polyclonal antibody, followed by a 1:2000 dilution of an anti-rabbit-HRP secondary antibody (Promega). Bands were visualized using the Opti-4CN detection system (BioRad). For all mutants, cleavage of P4a and P4b occurred at the permissive temperature but was absent or strongly reduced at the non-permissive temperature (Figure 3A). Cleavage of P25K at the AG*A site to produce 25 K did not occur at the non-permissive temperature, while a higher molecular weight band corresponding to the product created by cleavage at an AG*S site was present [17]. The banding patterns at the non-permissive temperature were similar to those seen in cells treated with rifampicin, a drug known to inhibit cleavage of core proteins [18]. Core protein precursor processing in both parental strains proceeded normally at 41C (data not shown).

The absence of cleavage at the non-permissive temperature was confirmed for P4a using pulse-chase immunoprecipitation. 100 mm plates of BSC_40 _cells were infected with virus at an MOI of 10 and incubated at 31 or 41C, as appropriate. At 8 hpi, the cells were labeled with 100 μCi of [^35^S]-methionine and [^35^S]-cysteine (EasyTag EXPRE^35^S^35^S; PerkinElmer) in methionine and cysteine free media. Rifampicin, where needed, was added at 100 μg/ml. After a 45 minute incubation, pulse wells were harvested, while chase wells were washed and treated with media containing a 100 fold excess of unlabeled methionine and cysteine and rifampicin if necessary. These were harvested at 24 hpi. Cell pellets were resuspended in 600 μl of RIPA buffer and subjected to three freeze/thaw cycles and sonication. Samples were centrifuged to remove debris and the lysate was incubated overnight with polyclonal antibodies against P4a followed by a second incubation after the addition of Protein A Sepharose beads (Amersham BioSciences). The washed beads were boiled in 20 μl of sample buffer and subjected to electrophoresis on a 4–12% SDS PAGE gel.

In all virus containing pulse samples, a strong band corresponding to P4a was clearly visible (Figure 3B). For IHD-W and Dts-4 at 31C, a representative example of the behavior of the ts viruses at the permissive temperature, the precursor containing band was strongly diminished after the chase period while a lower molecular weight band representing the cleaved product 4a appeared. At the non-permissive temperature, there was limited change in the intensity of the precursor and little or no cleavage product appeared. The pattern seen at the non-permissive temperature was similar to that of IHD-W infected cells treated with rifampicin.

**Figure 4:**
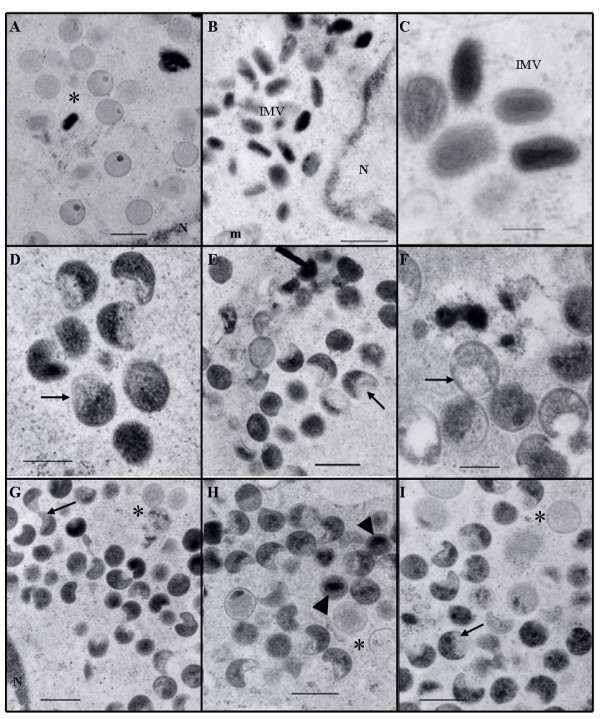
**Electron micrographs of virus infected BSC_40 _cells**. MOI = 10 and cells were harvested and fixed 24 hours after infection. Dts-4 at the permissive temperature of 31C (A-C). Dts-4 (D), Cts-16 (E), Cts-34 (F), Dts-8 (G), Dts-35 (H) and Dts-93 (I) at the non-permissive temperature of 41C. Bars represent 400 nm except in C, D and F (bar = 200 nm). N, nucleus; m, mitochondria; IMV, intracellular mature virion; asterisk, immature viral particle; arrow, representative particles with asymmetrical viroplasm condensation; arrow head, nucleoids.

The overall morphology of all six mutants was examined using electron microscopy. BSC_40 _cells were infected at an MOI of 10 and, after a one hour absorption period, incubated at 31 or 41C. Infected cells were collected at 24 hpi, fixed, embedded and stained. Dts-4 grown at 31C was examined as a representative of the ts mutants at the permissive temperature and was wild-type in its appearance. Both mature, brick-shaped particles with characteristic biconcave cores and spherical IV containing electron dense viroplasm were seen (Figure 4A–C). At the non-permissive temperature, all the ts mutants were similar in their microscopic appearance (Figure 4D–I). Normal crescent shaped membranes and IV were seen along with large numbers of defective IV. Many of the particles had asymmetrical condensation of the viroplasm, with the membrane sometimes collapsing on the empty side. Others formed dark, electron dense nucleoids. The appearance of these mutants is similar to what has previously been reported for Cts-16 [9,14], Dts-8 [13], and I7L conditional-lethals where I7L expression was inhibited by an operator/repressor system [6,8]. The appearance of Dts-35 differed from that reported by Dales [13], as particles with defective cores were not seen. However, Ansarah-Sobrinho and Moss also reported poorly formed cores in some of their I7L null mutants [6]. It seems then, that a deficiency in I7L can manifest itself in two different ways, with the virion morphology described here having been the most frequently observed.

Since the mutations in the I7L ts mutants fall into two distinct groups it is tempting to speculate that they might affect two different functions of I7L. Our results indicate that this is not the case, at least at the level of the virion formation, as all mutants were defective in the cleavage of core protein precursors and had their development arrested at a similar stage. Yet the possibility remains that the mutations affect two different elements required for proteinase function. The mutation in Cts-16 (and now Dts-8) at aa344 has been suspected, without proof, to inhibit protein cleavage by disrupting the arrangement of the catalytic triad due to its proximity to the cysteine residue at aa328. It is possible that the other mutants, with amino acid changes at the N-terminus of the protein between residues 29 and 37, may also sufficiently alter the structure or stability of the catalytic site to prevent proteolysis. However, because of their position this seems less likely. Instead, we suggest that the mutations occur within a region that constitutes a separate domain of unknown function that is necessary for I7L proteinase activity. Unfortunately the existing threading and homology model of I7L [15] does not include the 130 N-terminal most amino acids as this region does not fit any known structural domain.

Nevertheless, the properties of this region suggest several potential functions. One possibility is that the mutations disrupt the binding site of an unidentified co-factor(s) that I7L is believed to require as I7L produced in a cell-free translation system lacks cleavage activity [19]. The affected stretch of amino acids lies within a hydrophobic region [2], a common characteristic of sites of protein-protein interaction. The mutations also lie within a region that shows weak homology to the type II DNA topoisomerase of *Saccharomyces cerevisiae *[9], raising the possibility of a nucleic acid binding site. Adenovirus proteinase, an I7L homolog, requires both a peptide and a DNA cofactor for full activity [20]. Alternatively, the mutations may interfere with a potential regulatory cleavage as I7L contains two AG*X motifs at its N-terminal end and third at its C-terminal end. One of these AG*X sites is directly disrupted by the mutation in Cts-34 (AGL to AEL)

It is important to note that until more detailed structural and biochemical information about I7L is available, any conclusions about the processes disrupted by the mutations within these ts mutants are tentative. However, their location provides a starting point in the search for regions of I7L important to its activity.

## Competing interests

The author(s) declare that they have no competing interests.

## Authors' contributions

MJM conducted the experiments and wrote the manuscript. CMB assisted with the sequencing and edited the manuscript. DEH conceived the study, coordinated the research efforts and edited the manuscript. All authors read and approved the final manuscript.
